# Comparison of the efficacy of ultrasound-guided erector spinae plane block and thoracic paravertebral block combined with intercostal nerve block for pain management in video-assisted thoracoscopic surgery: a prospective, randomized, controlled clinical trial

**DOI:** 10.1186/s12871-022-01823-1

**Published:** 2022-09-10

**Authors:** Lingling Sun, Jing Mu, Bin Gao, Yuexian Pan, Lang Yu, Yang Liu, Huanzhong He

**Affiliations:** Department of Anesthesiology, Huzhou Central Hospital, 1558# Sanhuan North Road, Huzhou, 313000 China

**Keywords:** Postoperative analgesia, Video assisted thoracoscopic surgery, Erector spinae plane block, Thoracic paravertebral block, Intercostal nerve block

## Abstract

**Background:**

The objective of this study was to compare analgesic efficacy of erector spinae plane block(ESPB) and thoracic paravertebral block(TPVB) combined with intercostal nerve block(ICNB) after video assisted thoracoscopic surgery(VATS).

**Methods:**

Patients were enrolled into three groups according to analgesia technique as ICNB, TPVB + ICNB or ESPB + ICNB: respectively Group C(*n* = 58), Group T (*n* = 56) and Group E (*n *= 59). Patients were followed up by a trained data investigator at 2, 6, 8, 12, 24, 48 h after surgery, and the visual analog scale(VAS) at rest and coughing were recorded. The moderate and severe pain mean VAS ≥ 4 when coughing. The postoperative opioids consumption, incidence of postoperative nausea and vomiting (PONV), supplementary analgesic requirements within 48 h, length of stay in PACU, ambulation time, postoperative days in hospital and potential side effects, such as hematoma, hypotension, bradycardia, hypersomnia, uroschesis, pruritus and apnea were recorded.

**Results:**

The incidence of moderate-to-severe pain was no significant difference between 3 groups in 24 h and 48 h (*P* = 0.720). There was no significant difference among the 3 groups in the resting pain intensity at 2, 6, 8, 12, 24 and 48 h after surgery(*P* > 0.05). In 2-way analysis of variance, the VAS when coughing in Group T were lower than that in Group C (mean difference = 0.15, 95%CI, 0.02 to 0.29; *p* = 0.028). While no difference was found when comparing Group E with Group C or Group T(*P* > 0.05). There was no difference between the three groups in the sufentanil consumption( within 24 h *p* = 0.472, within 48 h *p* = 0.158) and supplementary analgesic requirements(*p* = 0.910). The incidence of PONV and the length of stay in PACU, ambulation time and postoperative days in hospital were comparable in the 3 groups(*P* > 0.05). Two patients from Group T developed hematoma at the site of puncture.

**Conclusions:**

The present randomized trial showed that the analgesic effect of TPVB + ICNB was superior to that of INCB after VATS, the analgesic effect of ESPB was equivalent to that of TPVB and ICNB.

**Trial registration:**

Chinese Clinical Trial Registry, ChiCTR2100049578. Registered 04 Aug 2020 Retrospectively registered.

## Background

Surgical resection remains one of the main methods for curative treatment of lung cancer in patients. Traditionally, resection is done via a thoracotomy, but video-assisted thoracoscopic surgery (VATS) provides significant advantages over open thoracotomy procedures including reduced surgical pain, improved post-operative pulmonary function, reduced mortality, shorten hospital stay and has emerged as a minimally invasive alternative [1–3]. Despite VATS association with lessened surgical trauma and better post-operative outcomes, a reduction in tissue damage did not necessarily lead to the same reduction in the need for analgesia. The intercostal nerve injuries, muscle injuries, rib contractions or even fractures and pleural lining damage all contribute to pain after thoracoscopic surgery. Controlling postoperative pain was crucial because increased acute pain has been related to the development of chronic pain, augmented respiratory complications, added hospital length of stay [4–8], and reduced patient satisfaction. Effective pain control would increase patients’ability for physiotherapy and pulmonary rehabilitation which could improve postoperative outcomes.

Thoracic epidural analgesia (TEA) once hailed as the gold standard of thoracotomy, is no longer the first choice for VATS because of the high potential risk associated with dural puncture, epidural hematoma, neuropathy and hypotension [9], stating that the peripheral blocks are taking place of the central blocks. However, the optimal analgesic choice for thoracoscopic surgery still needs to be identified. At present, the commonly used methods of regional nerve block include thoracic paravertebral block(TPVB), intercostal nerve block. These methods combined with patient-controlled intravenous drug-controlled analgesia have become the mainstream of postoperative analgesia after VATS [10–12], but every method has its own disadvantages. TPVB has recently been found to have similar pain control effects with fewer side effects than TEA [13]. However, it was not in regular practice because of technical challenge and potential risks (et.pneumothorax, blood vessel damage and so on) [14]. Thoracoscopic intercostal nerve block(ICNB) is widely used in VATS because of its technical safety and simplicity [15]. But the duration of action is limited, it necessitates multiple injections and large doses of NSAID are required to achieve the desired analgesic effect [10]. In 2016, Forero et al. proposed a relatively new technique called erector spinae plane block(ESPB) [16]. In the following years, more and more randomized controlled trials reported that ESPB may be used to provide effective analgesia management following VATS [17, 18].

Whether ICNB combined with TPNB or ICNB combined with ESPB can reduce more pain score after VATS is still unknown. This study was designed to compare the analgesic effects of ICNB combined with TPVB and ICNB combined with ESPB in patients undergoing thoracoscopic lobectomy.

## Material and methods

### Subjects and sample collection

After the study was approved by the Institutional Review Board and the protocol was registered (registration number:20180713–01), this randomized controlled trial was performed from January 2019 to December 2020 at the Huzhou Central Hospital in China. The study enrolled patients who were 18 to 80 years old, American Society of Anesthesiologists class I and II and scheduled for non-emergent lobectomy under VATS. Exclusion criteria were history of chronic pain or daily use of analgesics, history of psychiatric disorder or inability to understand the consent form or how to use a visual analog scale (VAS) for pain measurement, severe renal or hepatic dysfunction, allergy to any required drug, second thoracic surgery, participation in other clinical trials, obesity with body mass index > 35 kg/m^2^, intake of antiplatelet or anticoagulant agents, local infection at the injection site, spinal deformity and severe bradycardia. Patients were withdrawn from the study if technical failure happened in the block or VATS procedure was converted to open procedure.

All methods were performed in accordance with the relevant guidelines and regulations. All participants followed a standard perioperative care protocol. After the patient arrived at the outpatient anesthesia room, an investigator explained the details of the study protocol to the recruited patient and obtained the written informed consent. Anesthetic evaluation was performed by the anesthesia team, who were not aware of patient’s group assignment, and patients were instructed to use a 10-cm pain VAS (0 = no pain, 10 = worst imaginable pain). Then a SPSS generated random number table was used to allocate the patients into 1 of the 3 groups in a 1:1:1 ratio. Group C (the control group) received ICNB performed by the surgeon after induction of general anesthesia, and a total of 20 ml of 0.375% ropivacaine (5 ml per each space) was injected the lower margin of each intercostal upper rib avoids the intercostal vessels at T4-T7 levels under video guidance. Group T received unilateral, single-injection TPVB at T5 level with 20 ml of 0.375% ropivacaine after the patient was sedated with midazolam (0.04 mg/Kg) and ICNB after induction of general anesthesia. Similarly, Group E received unilateral, single-injection ESPB at T5 level with 20 ml of 0.375% ropivacaine after the patient was sedated with midazolam (0.04 mg/Kg) and ICNB after induction of general anesthesia. TPVB and ESPB were performed by the same anesthetist after being informed of the group allocation of the patient by a sealed opaque envelope and he was the only individuals aware of the treatment allocation, while patients and evaluators were blinded to the group assignment.

### Thoracic paravertebral block

**Fig. 1:**
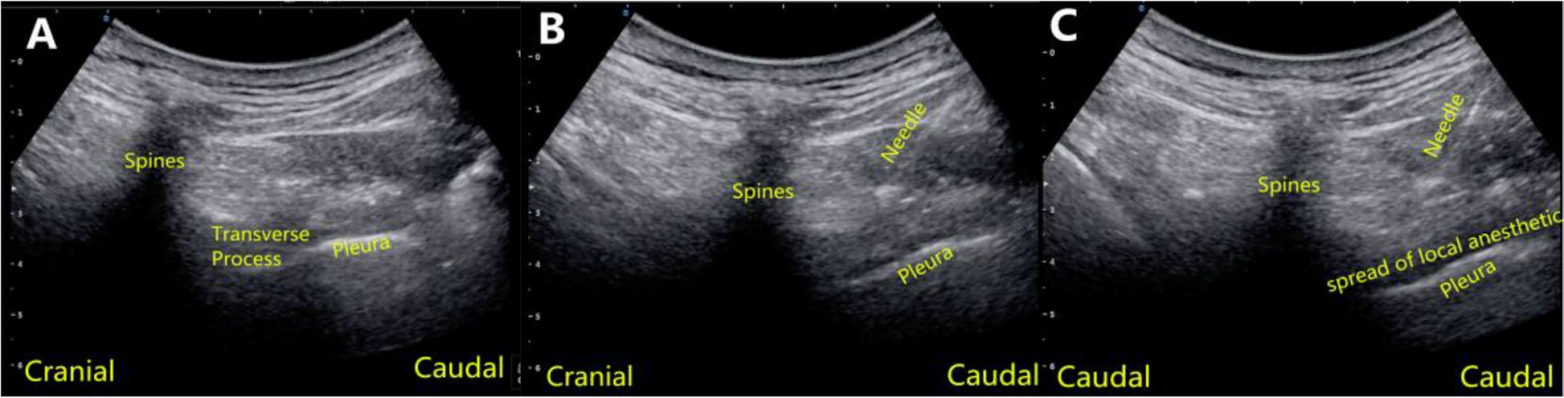
Paramidsagittal ultrasonography of T5 transverse process and thoracic paravertebral block performing figures. (**A**) Sonographic anatomy, (**B**) Needle direction, (**C**) Craniocaudal spread of local anesthetic

In the anesthesia preparation room, patients were monitored according to ASA standards and than sedated with midazolam (0.04 mg/Kg). An experienced anesthesiologist performed unilateral, single-injection TPVB at T5 level of the operation side with ultrasound guided (USG) in lateral position before anesthesia induction. A low-frequency convex array USG probe (2 to 5 MHz; Konica Minolta Sonimage HS1,Shanghai,China.) was placed longitudinally 2.5 cm lateral to the tip of spinous process to identify the hyperechoic image of pleura between shadows of consecutive transverse processes. Peripheral block needle (22 gauge; Stimuplex® A; B Braun, Melsungen, Germany) was advanced to the pleura using in-plane technique. After negative aspiration, downward displacement of the pleura by administration of saline was visualized on USG and then block was achieved with 20 ml of 0.375% ropivacaine. Local anesthetic distribution above pleura was checked by moving the probe up and down to confirm success of block (Fig. 1).

### Erector spinae plane block

**Fig. 2:**
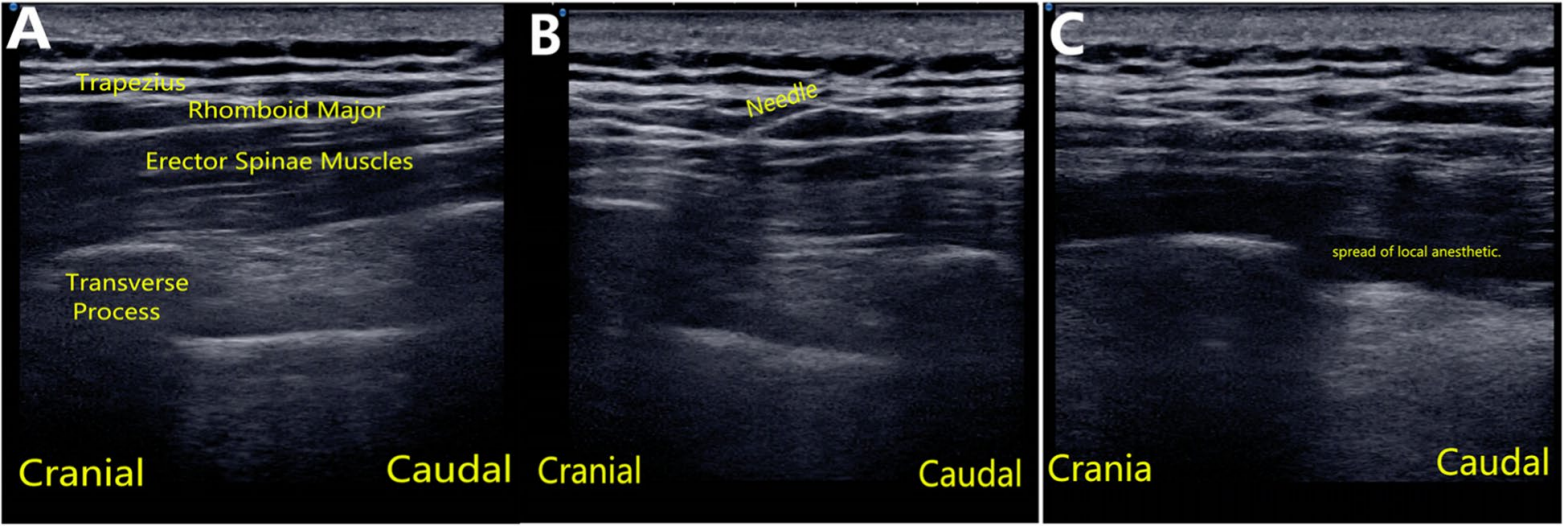
Paramidsagittal T5 transverse process ultrasonography and erector spinae plane block performing figure. (**A**) Sonographic anatomy, (**B**) Needle direction, (**C**) Craniocaudal spread of local anesthetic

Similar to TPVB procedure, patients were sedated after monitoring. The same anesthesiologist who applied TPVB performed unilateral single-injection ESPB at T5 level of the operation side with USG in lateral position before anesthesia induction. A high-frequency linear USG probe (5 to 13 MHz; Konica Minolta Sonimage HS1,Shanghai,China.) was placed longitudinally 2.5 cm lateral to the tip of spinous process to identify the trapezius, rhomboid major, and erector spinae muscles superficial to the hyperechoic transverse process shadow. Peripheral block needle (22 gauge; stimuplex D; B.Braun Melsungen AG, Melsungen, Germany)was inserted in the interfascial plane deep to the erector spinae muscle using in-plane technique. After negative aspiration, spread of saline in the interfacial plane was visualized on USG and then block was achieved with 20 mL of 0.375% ropivacaine. (The success of block was confirmed by USG. The probe was shifted over two upper (for T3, T4) and two lower (for T6, T7) transverse processes to check interfacial spread of local anesthetic at these levels (Fig. 2).

During the operation, standardized monitoring was applied. Anesthesia was induced with propofol (1.5 mg/kg), sufentanil (0.5ug/kg), and rocuronium(0.6 mg/kg) was administered to facilitate left double lumen tube (DLT) intubation. The correct position of DLT was confirmed with a fiberoptic bronchoscope. After tracheal intubation, a volume-cycled ventilator was applied with the following settings: tidal volume of 6-8 ml/kg ideal body weight and inspiratory-to-expiratory ratio of 1:2 were used for double lung ventilation. While during single-lung ventilation, tidal volume of 4-6 ml/kg and inspiratory-to-expiratory ratio of 1:2 was set. The petCO_2_ was 35-45 mmHg(1 mmHg = 0.133kpa) maintained by intraoperative regulation of respiratory rate, and oxygen saturation > 95% was maintained by regulation of oxygen concentration (0.6–1). Anesthesia maintenance was achieved with sevoflurane inhalation, continuous infusion of propofol and remifentanil to maintain the bispectral index 40 to 60. Intraoperative hypotension (defined as decrease of 20% from baseline value or mean arterial pressure less than 65 mmHg) was treated with noradrenaline infusion, and bradycardia (heart rate < 50 beat per minute) was treated with atropine. The lobectomy procedure was performed by the same surgical team. All patients underwent lobectomy in lateral decubitus position. At the end of the surgery, chest tube was placed through the seventh intercostal space as required.

At the end of surgery, all patients received intravenous tramadol 100 mg as loading dose for analgesia. According to our clinical routine postoperative analgesia scheme, each patient received intercostal nerve block by the surgeon combined with patient-controlled analgesia(PCA) with opioids. ICNB was performed at the beginning of surgery, and a total of 20 ml of 0.375% ropivacaine (5 ml per each space) was injected at T4-T7 levels under video guidance. The PCA protocol was 0.1 mg of sufentanil diluted to 100 ml with a continuous dose of 0.03–0.05 ml•kg − 1•h − 1 and a bolus dose of 0.02–0.03 mL•kg − 1 with a lock-out of 15 min. PCA device was attached to the patient immediately after surgery and was stopped after 48 h. Group T and Group E received preoperative TPVB or ESPB respectively as demonstrated previously. If the analgesia was inadequate (visual analog scale, VAS ≥ 4) during the postoperative period, patients were recommended to press the PCA button. And if relief was not obtained, additional analgesics (40 mg Parecoxib sodium) were given intravenously as rescue, and consultation with the anesthetist was initiated as required. All patients received granisetron(3 mg) at the end of surgery to prevent postoperative nausea and vomiting, and additional antiemetic was given as rescue if vomiting occurred or if persistent nausea was reported for 2 h. Patients were transferred to post-anesthesia care unit (PACU) after tracheal extubation and discharged from PACU when Aldrete score reached 8 [19].

Patients were followed up by a trained data investigator at 2, 6, 8, 12, 24, 48 h after surgery, and the VAS at rest and coughing were recorded. The primary outcome was the proportion of patients suffering moderate-to-severe pain (VAS ≥ 4 when coughing) at 24 h after surgery. Secondary outcomes included VAS at rest and VAS when coughing at each time point, postoperative opioids consumption, incidence of postoperative nausea and vomiting, supplementary analgesic requirements within 48 h, length of stay in PACU, ambulation time and postoperative days in hospital. Potential side effects, such as hematoma, hypotension, bradycardia, hypersomnia, uroschesis, pruritus and apnea were recorded.

### Data processing and statistical analysis

A previous study reported that the incidence of moderate-severe pain following VATS was 59% [20]. Assuming a decrease of 25% as the minimally clinical significance, 50 patients were needed per study group with a two-tailed power analysis with 0.90 power and an α of 0.05. Considering a possible dropout, a total of 180 patients were required in the study so as to fit the analysis of variance models and to allow for comparisons among other outcome variables of interest.

All statistical data was analyzed via SPSS version 17.0 (SPSS Inc., Chicago, IL, USA). The Kolmogorov–Smirnov test was used to determine the normality of data distribution. Continuous variables were expressed as mean ± standard deviation, or median (interquartile range). Categorical variables were expressed as number (percentage). One-way ANOVA was used for normally distributed continuous variables, while Kruskal–Wallis test was used for non-normally distributed continuous variables to analyze the differences among groups, followed by Dunn’s multiple comparison tests. Two-way analysis of variance with Bonferroni post hoc test was used to evaluate the effect of the intervention and the assessment timepoint. Comparisons of categorical variables between the groups were performed using Chi-Square test or Fisher test as appropriate. *P* < 0.05 was considered statistically significant.

## Results

**Fig. 3:**
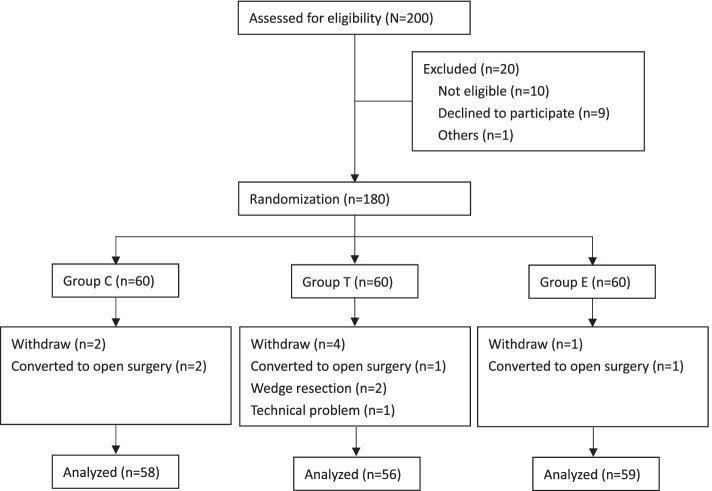
Flowsheet

During the study period, 200 patients were assessed for eligibility. After exclusion of 20 patients, 180 patients were randomized into 3 groups with 60 patients per group. A total of 7 patients were withdrawn by the end of the trial due to 4 patients conversion to open surgery (*n* = 4), 2 patients underwent wedge resection rather than lobectomy (*n* = 2) and technical problem (*n* = 1), leaving 173 patients for analysis (Fig. 3). The average age of the study population was 57.0 ± 11.4 years and 61.3% of study participants were female. There was no significant difference among the 3 groups in demography and intraoperative characteristics, including age, gender, weight, BMI, American Society of Anesthesiologists classification, smoking history, surgery duration and chest tube placed (*P *> 0.05) (Table 1).

**Fig. 4:**
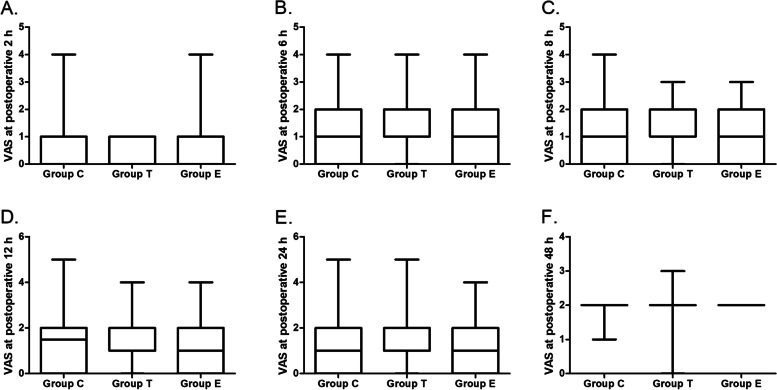
The dynamic changes of resting pain intensity evaluated by visual analog scale at postoperative 2 (**A**), 6 (**B**), 8 (**C**), 12 (**D**), 24 (**E**) and 48 h (**F**). Boxes represent first/third quartile, lines represent medians, and whiskers represent the minimum and maximum values

**Table 1:** Demographic and intraoperative characteristics of patients in the 3 groups

	Group C (*n *= 58)	Group T (*n *= 56)	Group E (*n *= 59)	P
Age	56.5 ± 12.6	59.0 ± 11.2	55.5 ± 10.2	0.251
Gender				0.505
Male (n, %)	26 (44.8%)	20 (35.7%)	21 (35.6%)	
Female (n, %)	32 (55.2%)	36 (64.3%)	38 (64.4%)	
Weight (kg)	61.8 ± 10.2	59.4 ± 8.0	60.4 ± 10.9	0.431
BMI (kg/m2)	22.9 ± 2.9	22.9 ± 2.9	22.6 ± 2.9	0.873
ASA				0.936
II	52 (89.7%)	51 (91.1%)	54 (91.5%)	
I	6 (10.3%)	5 (8.9%)	5 (8.5%)	
Smoking history (n, %)	24(41.4%)	19(32.2%)	19(33.9%)	0.548
Duration of surgery (min)	136.4 ± 57.8	150.6 ± 53.4	131.3 ± 60.2	0.189
Chest tube (n, %)	55( 94.8%)	54( 96.4%)	52(88.1%)	0.175

The incidence of moderate-to-severe pain(VAS ≥ 4 when coughing) within postoperative 24 h in Group C, Group T and Group E were 37.9%, 37.5% and 44.1%, respectively. There was no significant difference between 3 groups (*P* = 0.720). Similarly, the incidence of moderate-to-severe pain within 48 h in the 3 groups were also comparable (37.9% in Group C,37.5% in Group T and 44.1% in Group E).

**Fig. 5:**
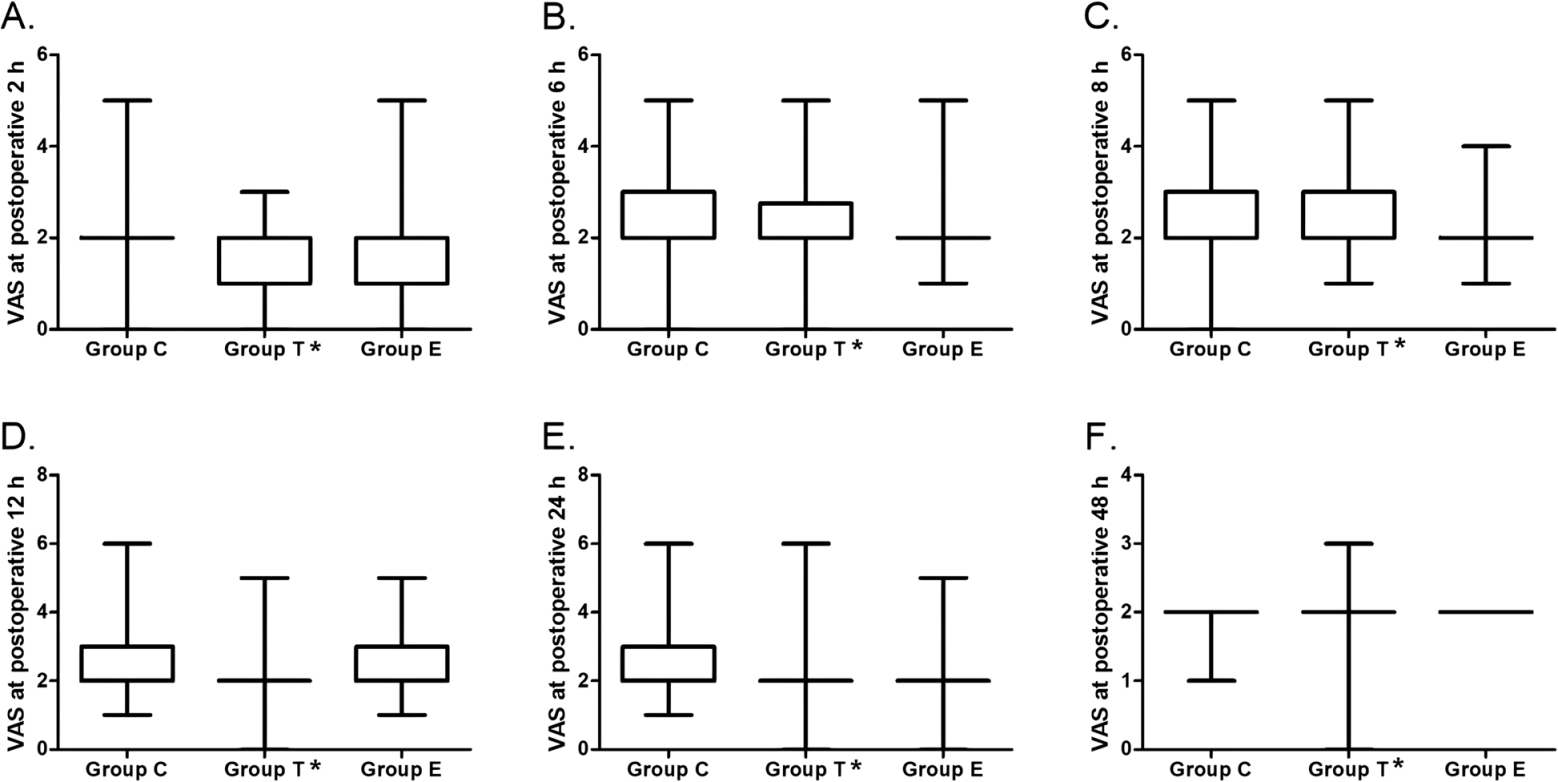
The dynamic changes of pain intensity when coughing at postoperative 2 (**A**), 6 (**B**), 8 (**C**), 12 (**D**), 24 (**E**) and 48 h (**F**). *Compared with group C, P < 0.05. Boxes represent first/third quartile, lines represent medians, and whiskers represent the minimum and maximum values

There was no significant difference among the 3 groups in the resting pain intensity at 2, 6, 8, 12, 24 and 48 h after surgery (Fig. 4). In 2-way analysis of variance, the VAS when coughing in Group T were lower than that in Group C (mean difference = 0.15, 95%CI, 0.02 to 0.29; *p* = 0.028). While no difference was found when comparing Group E with Group C or Group T (Fig. 5).

The sufentanil consumption and supplementary analgesic requirements were comparable in the 3 groups. A total of 17 patients (9.8%) required supplementary analgesic, and 19 patients (11.0%) experienced PONV within 48 h. There was no difference in the incidence of PONV and supplementary analgesic requirements within 48 h postoperatively. The length of stay in PACU, ambulation time and postoperative days in hospital were similar in the 3 groups. Two patients from Group T developed hematoma at the site of puncture, but there was no difference in the incidence of hematoma among the three groups (Table 2). And no other side effects was observed during the study period.

**Table 2:** Comparison of secondary outcomes among the 3 groups

	Group C (*n *= 58)	Group T (*n* = 56)	Group E (*n *= 59)	P
Sulfentanyl consumption (ug)				
Within 24 h	43.7 ± 6.7	45.6 ± 8.7	43.7 ± 12.0	0.472
Within 48 h	99.3 ± 3.4	96.7 ± 8.2	97.0 ± 9.8	0.158
PONV within 48 h (n, %)	6 (10.5%)	8 (14.3%)	5 (8.5%)	0.603
Supplementary analgesic requirements (n, %)	6 (10.3%)	6 (10.7%)	5 (8.5%)	0.910
Length of stay in PACU (min)	58.6 ± 19.4	53.4 ± 10.0	57.7 ± 14.3	0.149
Ambulation time (hours)	29.3 ± 15.2	29.3 ± 24.1	33.8 ± 26.4	0.464
Postoperative days in hospital (days)	5.3 ± 2.7	5.6 ± 2.4	4.7 ± 1.6	0.116
Incidence of hematoma(n,%)	0(0%)	2(3.6%)	0(0%)	0.121

## Discussion

We compared analgesic efficacy of TPVB + ICNB, ESPB + ICNB and ICNB alone for thoracoscopic lobectomy in this study, and all the three blocks guaranteed adequate pain control. The incidence of moderate to severe pain within 24 h after surgery was similar in all three groups. Our study has shown that US-guided unilateral single shot ESPB or TVPB performed before general anesthesia induction in VATS patients was the same in the resting pain intensity at 2, 6, 8, 12, 24 and 48 h after surgery when compared to that in control group. The VAS when coughing in TPVB + ICNB were lower than that in ICNB. Whereas ESPB showed similarly pain scores when compared to ICNB or TPVB in cough. Postoperative opioid consumption was similar in all three groups. TPVB + ICNB may seems to be a better method with a more successful analgesia compared to ICNB alone in thoracoscopic surgery.

Video-assisted thoracoscopic surgery was related to reduce pain, lung function protection, faster recovery, shorter hospital stay, and better quality of life. In addition, the advantages of thoracoscopic approach for early lung cancer were also reported in ERAS guidelines [21]. Whereas, it still cause significant acute pain after surgery and it may even lead to neuropathic pain syndrome. Therefore, a multimodal approach to opioid retention such as TPVB, ESPB and ICNB is strongly recommended. TPVB is a method to block the movement, sensation and sympathetic nerve of the side by injecting local anesthesia near the spinal nerve of the intervertebral foramen to achieve the analgesic effect of ipsilateral body. Performing a single shot block of TPVB may can achieve unilateral epidural block effect, but it was certain technical challenges and risk of pneumothorax, hematoma and spinal cord injury. The thoracic nerve immediately enters the paraspinal space after exiting the foramina, but there is no direct connectivity between the intervertebral spaces, so it is often necessary to use multiple TPVB to effectively inhibit perioperative stress response and pain. The intercostal nerves come from the anterior branch of the thoracic paravertebral nerve, it was performed primarily by surgeons at multiple levels of direct visualization. The block was considered easy, with relatively low side effects and have been used as an analgesic replacement to TPVB in VATS with beneficial effect [22]. Although ICNB has shown to lower pain scores in the early postoperative period, but its duration of action was limited. ESPB was an interesting alternative to thoracic wall block and may have similar effects to TPVB [16, 23]. ESPB does not involve the thoracic nerve root through paravertebral insertion, which has the advantage of being distant from the pleura and neural structures. This may mean decreasing the risk of pneumothorax and hematoma, which makes ESPB safer than central blocks including TPVB and preferable for patients on anticoagulant therapy. On magnetic resonance imaging, ESPB showed that the injection spread over 2–5 segments from the epidural and internerve foramen to more than 5–9 segments from the intercostal space [24, 25]. Local anesthetic injection into the spinal plane of the vertical spine can be longitudinally diffused through the thoracolumbar fascia to the cephalic and caudal spaces of the thoracic paravertebral space to achieve the effect of blocking the thoracic wall nerves and visceral nerves simultaneously. The spread of ESPB was unpredictable and it was discussed as a limitation on experimental, cadaveric or volunteers studies [26, 27]. These blocks are important component of multimodal analgesia for thoracic surgery [28]. The three regional block techniques have their own advantages and disadvantages. Their combined use may achieve better analgesic effect and reduce complications, and provide new ideas for optimal postoperative analgesia.

A study comparing these three blocks for thoracoscopic surgery indicated TPVB was superior to two others with effective pain control in all study groups, while they used multilayer injection (T5, T6 and T7) technique for TPVB [29], we preferred a single level approach. Another study comparing these three blocks showed that TPVB reveals to be a superior approach compared to ESPB and ICNB with more successful analgesic effect and less remedial analgesic during the first 24 h of VATS, whereas ICNB showed the same low pain scores compared to ESPB [30]. Their blocking techniques are used independently. We found that TPVB and ESPB provided a comparable amount of pain relief during the first 24 h. Our results confirmed the findings of previous studies [31] showed that the analgesic effect of ESPB was non-inferior to that of TPVB within 24 h after VATS. We used TPVB and ESPB combination with INCB. In fact, the efficacy of postoperative analgesic strategies should be evaluated in various aspects, and not only treatment of acute pain.

In our study we used an intravenous sufentanil PCA and regional nerve block to manage postoperative analgesia following thoracoscopic surgery, and all the three groups guaranteed adequate pain control. Intravenous patient controlled analgesia is one of strictly recommended by international guidelines for pain management in thoracic surgery [32]. An observational study showed the efficacy and safety of sufentanil sublingual tablet system to manage postoperative analgesia following thoracic surgery [33]. In that study, pain management for all patients is only sufentanil sublingual tablet system, and patients experienced moderate to severe pain with a score around 5 at rest and around 7 at cough in PACU. Patients had a mean pain score of less than 3 at 6 h of rest and at 36 h of cough after surgery. This would be a terrifying experience for patients. While, the system was safty and not invasive, and patients benefited rapidly after taking tablets, as pain scores decreased rapidly to mild pain. This suggests that the system will be effective in dealing with acute pain. It provides a new idea for postoperative analgesia after thoracic surgery, which can be used as a remedial analgesic measure.

There was no difference in the postoperative sufentanil consumption, incidence of PONV and supplementary analgesic requirements within 48 h postoperatively. The length of stay in PACU, ambulation time and postoperative days in hospital were similar in the 3 groups. Other issue was that 2 patients had hematoma for TPVB at the site of puncture, but there was no difference in the incidence of hematoma among the three groups. The operation of TPVB is difficult due to its proximity to important anatomical structures such as pleura and central nervous axon. The anatomical structure is also difficult to be clearly recognized under the guidance of US. Therefore, it may cause complications, such as hematoma, pneumothorax or neuraxial injury. The advantage of US-guided ESPB was that it located further away from the pleura and neurologic structures, meanwhile the three layers of muscle structures are easily identified on ultrasound images. In addition, the hematoma was excluded in our study by direct video observation of ICNB. Nevertheless, the significant differences may need to be confirmed in large clinical studies.

### Limitations

First, our postoperative follow-up was up to 48 h and we did not investigate chronic pain. Second, since nerve blocks as part of the multimodal analgesia program, the analgesic differences between the two blocks might have been masked to some extent by the other analgesia, such as intercostal nerve block. Thirdly, we used 20 mL of 0.375% ropivacaine, but we did not evaluate different volumes or concentrations. These parameters should be evaluated in future research.

## Conclusions

The analgesic effect TPVB + ICNB was superior to ICNB alone when coughing in the postoperative period of VATS. The postoperative analgesia is similar when ESPB and TPVB, ESPB + ICNB and ICNB were compared in VATS. In conclusion, TPVB + ICNB may appears to be the preferable method with a more successful analgesia compared to ICNB alone in thoracoscopic lobectomy.

## Data Availability

The datasets used and/or analysed during the current study are available from the corresponding author on reasonable request.

## Abbreviations

VATS: Video-assisted thoracoscopic surgery
TEA: Thoracic epidural analgesia
TPVB: Thoracic paravertebral block
ICNB: Thoracoscopic intercostal nerve block
NSAID: Nonsteroidal antiinflammatory drugs
ESPB: Erector spinae plane block
PACU: Post-anaesthesia care unit
VAS: Visual analog scale
mL: Millilitres
mg/Kg: Milligram per kilogram
ug/kg: Microgram per kilogram
DLT: Double lumen tube
PCA: Patient-controlled analgesia
ml/Kg: Millilitres per kilogram
ml/Kg/h: Millilitres per kilogram per hour
BMI: Body mass index
ERAS: Enhanced Recovery After Surgery
